# Supplementary material for: The interactome of intact mitochondria by cross-linking mass spectrometry provides evidence for coexisting respiratory supercomplexes
Source: Mol Cell Proteomics. 2017 Dec 8;17(2):216–32. doi: 10.1074/mcp.RA117.000470 (PMC5795388; doi:10.1074/mcp.RA117.000470)
Supplement: Supplemental Data [file supp_RA117.000470_133922_0_supp_23978_fzffwf.zip › spectra_annotation/mito_spectra_annotation/252-1-10-1-10-1.pdf]

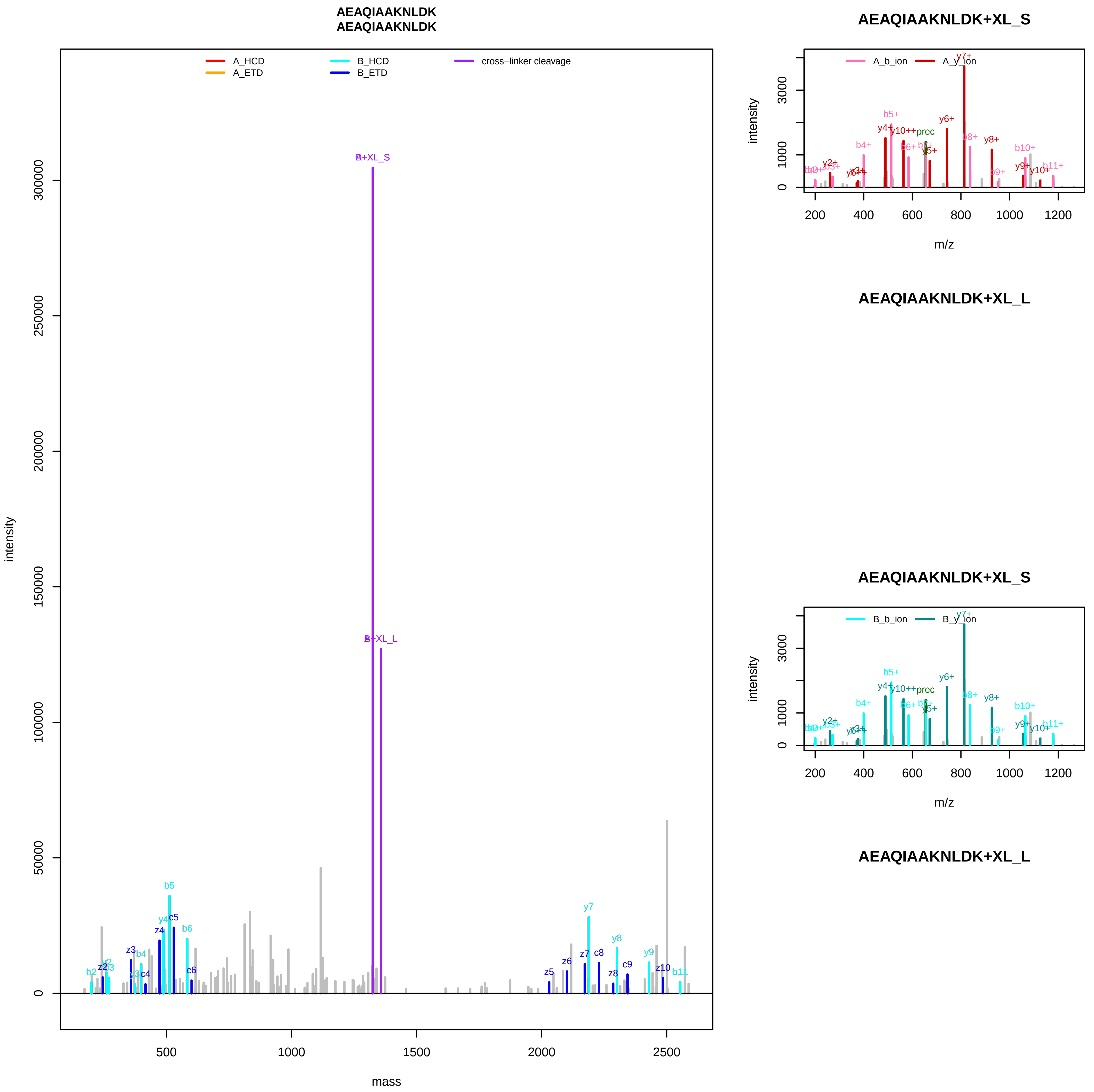

AEAQIAAKNLDK
AEAQIAAKNLDK
A_HCD
A_ETD
B_HCD
B_ETD
cross−linker cleavage
B+XL_S
B+XL_L
b2
z2
y2
z3
y3
b4
c4
z4
y4
b5
c5
b6
c6
z5
z6
z7
y7
c8
z8
y8
c9
y9
z10
b11
intensity
mass
0
50000
100000
150000
200000
250000
300000
500
1000
1500
2000
2500
AEAQIAAKNLDK+XL_S
A_b_ion
A_y_ion
B_b_ion
B_y_ion
prec
m/z
200
400
600
800
1000
1200
0
1000
3000
AEAQIAAKNLDK+XL_L
